# Demographic disparities in access to COVID-19 clinical trial sites across the United States: a geospatial analysis

**DOI:** 10.1186/s12939-024-02360-8

**Published:** 2025-01-23

**Authors:** Raphael Cuomo, Tiana McMann, Qing Xu, Zhuoran Li, Joshua Yang, Julie Hsieh, Christine Lee, Milena Lolic, Richardae Araojo, Tim Mackey

**Affiliations:** 1 School of Medicine, University of California, 8950 Villa La Jolla Drive, A203, San Diego, CA 92037 USA; 2 Global Health Policy and Data Institute, San Diego, CA USA; 3 Department of Anthropology, University of California, San Diego, CA USA; 4 S-3 Research, San Diego, CA USA; 5 San Diego Supercomputer Center, San Diego, CA USA; 6 Department of Public Health, California State University, Fullerton, Fullerton, CA USA; 7 Office of Minority Health and Health Equity, U.S. Food and Drug Administration, Silver Spring, MD USA

**Keywords:** COVID-19, Geospatial analysis, Demographics, Health equity, Clinical trials

## Abstract

Throughout the COVID-19 pandemic, underserved populations, such as racial and ethnic minority communities, were disproportionately impacted by illness and death. Ensuring people from diverse backgrounds have the ability to participate in clinical trials is key to advancing health equity. We sought to analyze the spatial variability in locations of COVID-19 trials sites and to test associations with demographic correlates. All available and searchable COVID-19 studies listed on ClinicalTrials.gov until 04/04/2022 and conducted in the United States were extracted at the trial-level, and locations were geocoded using the Microsoft Bing API. Publicly available demographic data were available at the county level for national analysis and the census tract level for local analysis. Independent variables included eight racial and ethnic covariates, both sexes, and twelve age categories, all of which were population-normalized. The county-level, population-normalized count of study site locations, by type, was used as the outcome for national analysis, thereby enabling the determination of demographic associations with geospatial availability to enroll as a participant in a COVID-19 study. Z-scores of the Getis-Ord Gi statistic were used as the outcome for local analysis in order to account for areas close to those with clinical study sites. For both national (*p* < 0.001) and local analysis (*p* = 0.006 for Los Angeles, *p* = 0.030 for New York), areas with greater proportions of men had significantly fewer studies. Sites were more likely to be found in counties with higher proportions of Asian (*p* < 0.001) and American Indian or Alaska Native residents (*p* < 0.001). Areas with greater concentrations of Black or African American residents had significantly lower concentrations of observational (*p* < 0.001) and government-sponsored COVID-19 studies (*p* = 0.003) in national analysis and significantly fewer concentrations of study sites in both Los Angeles (*p* < 0.001) and New York (*p* = 0.007). Though there appear to be a large number of COVID-19 studies that commenced in the US, they are distributed unevenly, both nationally and locally.

## Background

The coronavirus disease (COVID-19) was declared a public health emergency of international concern by the World Health Organization at the end of January 2020 and to date has claimed more than 6.2 million lives globally, with more than 1 million lives lost in the United States alone [1, 2]. Critical to arresting the spread of this devastating global pandemic has been investment and rapid research and development (R&D) of vaccines, therapeutics, diagnostics, and other medical countermeasures (MCMs) [3]. Robust public and private partnership on MCM R&D has led to the introduction of new vaccine platforms, oral antivirals and monoclonal antibodies, and rapid diagnostics tests, all of which have significantly reduced COVID-19-related morbidity and mortality [4–6]. As of December 2022, there were a total of 436,584 studies registered globally in ClinicalTrials.gov (“CT.gov”, a U.S. government sponsored database of privately and publicly funded clinical studies).

The need to develop MCMs as a key public health strategy to mitigate the social, health, and economic consequences of the COVID-19 pandemic has also increased urgency around ensuring that clinical research purposefully addresses known and widening health equity concerns, including ensuring inclusive and diverse trial demographics and participation [7–10]. Throughout the COVID-19 pandemic, underserved populations, such as racial and ethnic minorities, were disproportionately impacted by illness and death. Racial and ethnic minority groups have experienced mortality rates 10–50% higher than non-Hispanic Whites [11, 12]. Participants in clinical trials should reflect the individuals who may one day need to rely on these drugs, vaccines or devices to manage illness. Ensuring people from diverse backgrounds have the ability to participate in clinical trials is key to advancing health equity and advancement of science. Though prior studies have been conducted to analyze characteristics of clinical studies using ClinicalTrials.gov (“CT.gov”) for various medical conditions, no study we are aware of has looked specifically at demographic variables in spatial proximity to COVID-19 studies [13–16]. To remediate this existing gap, this study aims to characterize the spatial variability of U.S.-based COVID-19 clinical trial sites and to assess their geographic associations with demographic variables, thereby enabling the identification of communities that may have disproportionately low access to COVID-19 studies. Therefore, this study seeks to promote better understanding of COVID-19 and clinical trial-related health disparities research by understanding how geographic patterns to trial access may vary at the local and national level.

## Methods

This analysis assessed the spatial clustering of COVID-19 study site locations, by type, in the United States as well in select localities where significant hot spot clusters of study sites were detected. In this paper, we refer to “study sites” as spatial locations listed on CT.gov where COVID-19 research was being conducted. Data collection involved: (a) extraction and data mining of the location and characteristics of COVID-19 studies from CT.gov; (b) de-aggregation of studies by location, for multi-site studies; (c) geocoding of locations to produce latitude and longitude coordinates; and (d) aggregation of study locations to county and census tract bins. Data analysis involved: (i) computation of the Getis Ord Gi* statistic for spatial clustering; (ii) normalization using county-level population for national analysis and census tract-level population for local analysis; and (iii) assessing the relationship between area clustering and demographic composition with other secondary data related to race and ethnicity, sex, and age.

### Data collection

Data on clinical studies categorized as associated with “COVID-19,” “COVID-19 acute respiratory distress syndrome,” “COVID-19 Lower Respiratory Infection,” “COVID-19 Pandemic,” “COVID-19 Pneumonia,” “COVID-19 Respiratory Infection,” or “Coronavirus” were collected from CT.gov on April 4, 2022 using data extraction tools available on the website. No date restrictions were placed on the extraction. A manual review of study titles suggested that the study categories had face validity for studies addressing infection with the SARS-CoV2 pathogen. The export included one row per clinical study, with a location covariate which had, for each study, an array of study sites. A script was written in the Python programming language to generate a dataset whereby cases corresponded to distinct study sites, rather than studies themselves. Data on specific study characteristics were attributed to each study site. Other than location, covariates for study characteristics included enrollment for the overall study, study start date, study completion date (if available), funding source, intervention type, trial phase, sponsor name, and study title. As part of feature engineering, functions in Microsoft Excel were used to search strings in covariates from CT.gov. Specifically, intervention data were searched for terms “drug,” “biological,” and “behavioral.” Funding source categorizations made available from CT.gov were predominantly “NIH,” “Industry,” “Other,” or some combination of these three (e.g. “Industry|Other” or“Other|NIH”). Data on trial phase was also converted into four binary variables (one for each phase) to enable statistical analysis. Data on county-level and census tract-level demographic composition were obtained from five-year estimates of the U.S. Census Bureau’s American Community Survey (ACS) for calendar year 2020. Data on urban and rural categorization were taken from metropolitan classifications available from the U.S. Department of Agriculture (USDA).

Latitude and longitude coordinates for study locations were obtained for each study site using the Bing Maps Application Programming Interface (API). Coordinates for study sites were then plotted on a Global Coordinate System using the 1984 World Geodetic System on ArcGIS Desktop version 10.7. All study sites were matched to latitude and longitude coordinates, and mapped locations were manually reviewed in instances where location text from CT.gov appeared ambiguous (e.g. not an address or landmark). The count of study sites was computed for each U.S. county, as well as for each U.S. census tract, and then used to create geodatabases with county- and tract-specific data for population demographics.

### Data analysis

For descriptive analysis of U.S.-based trial sites, percentages were calculated to assess the distribution of all COVID-19 studies by funding source type, study length, and intervention/observational study type. For interventional studies, the proportion of trials addressing drugs, biologics, and behaviors were computed, as well as the proportion of trials by each phase. Total enrollment and average enrollment were computed for each stratum of descriptive categorization. Descriptive analysis was conducted at the study level rather than the site level, as information from CT.gov was attributable to the overall study rather than the individual sites.

For each polygon, the Getis Ord Gi* statistic was computed based upon the aggregation of each of the following nine types of studies and different categories: A) all COVID-19 studies, (B) phase 1 trials, (C) phase 2 trials, (D) phase 3 trials, (E) phase 4 trials, (F) interventional studies, (G) observational studies, (H) government-sponsored studies, and (I) industry-sponsored studies. These nine analyses were conducted at the national level and for each of the local sites included in this study as detected by our hot spot analysis (see below). The Getis-Ord Gi* statistic evaluates each feature in the dataset, considering its value as well as the values of its neighboring features, within the context of the entire dataset. This approach identifies clusters, or "hot spots," where a feature (e.g., a county) and its nearby neighbors have significantly higher values compared to the dataset as a whole. The fixed distance band was used as the weight matrix, enabling neighboring polygon counts to factor into the calculation of Getis Ord Gi* for the given polygon. Getis Ord Gi* statistics were used to produce a *p* value for statistically significant hot spots (clustering of high values) and cold spots (clustering of low values) for each polygon. Visualizations of statistically significant clustering were then generated.

For comparative national analysis, population-normalized counts of study sites, by type, as well as aggregated study enrollment, were used as outcome variables for statistical analysis. For local analysis, to account for the relevance of census tracts spatially proximal to those with study sites, *z*-scores of the Getis Ord Gi* statistic for total counts was used as the outcome variable to represent possible association with study access. This approach allowed local analysis to involve a dependent variable which exhibited a gradient for clustering, enabling empty tracts neighboring those with study sites to exhibit non-zero values and therefore permitting a more valid measure of study site availability. Independent variables for all analyses were also population-normalized for race and ethnicity categories, sex, and age group. Statistical software excluded non-Hispanic White race and age group 65–74 years from multivariable modeling due to multicollinearity. Regression analysis was conducted using R version 4.1.2.

## Results

### Clinical trial characteristics

There were a total of 1,653 COVID-19-related studies registered on CT.gov as of April 4, 2022. Of these, 13.2% (*n* = 219) were government-sponsored, 36.7% (*n* = 585) were industry sponsored, and the remainder 51.4% (*n* = 849) were categorized as "Other." Additionally, 70.0% (*n* = 1155) were categorized as interventional and 29.0% (*n* = 479) were observational. Furthermore, 85.2% (*n* = 1409) of studies enrolled only participants at least 18 years of age. Study start dates were skewed toward earlier time frames, with 948 (58%) starting in 2020, 549 (37%) starting in 2021, just 107 (7%) starting in 2022 (prior to the April 4th data collection date). The median study length was 392 days. However, the completion dates of 743 studies (45.5%) in our sample were after the date when we collected these data (04–04–2022). Nearly all study sites (96.8%) were located in urban areas.

Of the 1,155 trials categorized as interventional, 51.8% (*n* = 599) studied drug-based interventions, whereas only 21.9% (*n* = 253) were for biologics and 15.7% (*n* = 182) for behavioral interventions (see Table 1). Aggregate reported enrollment was 724,838 for drug-based studies (x̄ = 1,210), 771,275 for biologics-based studies (x̄ = 3,049), and 2,646,639 for behavior-based studies (x̄ = 14,542). An outlier was removed for a behavioral trial with projected enrollment of 40,000,000. The count of trials by phase appeared to be approximately normally distributed.

**Table 1:** Descriptive statistics for COVID-19 studies in the United States registered on clinicaltrials.gov

Categories	Total Enrollment	Average Enrollment	Frequency	Percentage of *COVID-19* Trials
NIH/Govt-Sponsored	42,272,220	193,024	219	13.2%
Industry-Sponsored	52,165,038	89,018	585	35.4%
Other	17,207,228	20,268	849	51.4%
Interventional	44,616,598	38,595	1,155	69.9%
Observational	67,027,888	136,513	479	29.0%
Other	0	0	19	1.1%
**Interventional Study Categories**	**Total Enrollment**	**Average Enrollment**	**Frequency**	**Percentage of *Interventional* Trials**^**a**^
Drugs	724,838	1,210	599	51.8%
Biologics	771,275	3,049	253	21.9%
Behavioral	2,646,639	14,542	182	15.7%
Other	1,252,739	5,447	230	20.0%
Early Phase 1	5,191	192	27	2.3%
Phase 1	6,162	53	116	10.0%
Phase 1|Phase 2	14,083	231	61	5.3%
Phase 2	267,949	797	336	29.1%
Phase 2|Phase 3	164,032	2,546	62	5.4%
Phase 3	444,117	3,218	138	12.0%
Phase 4	58,091	1,139	51	4.4%
Not Applicable	3,656,973	9,523	364	31.6%

^a^Categories for intervention type may overlap, as some trials were designated as studying multiple interventions

### Geographic characteristics of trial locations

**Fig. 1:**
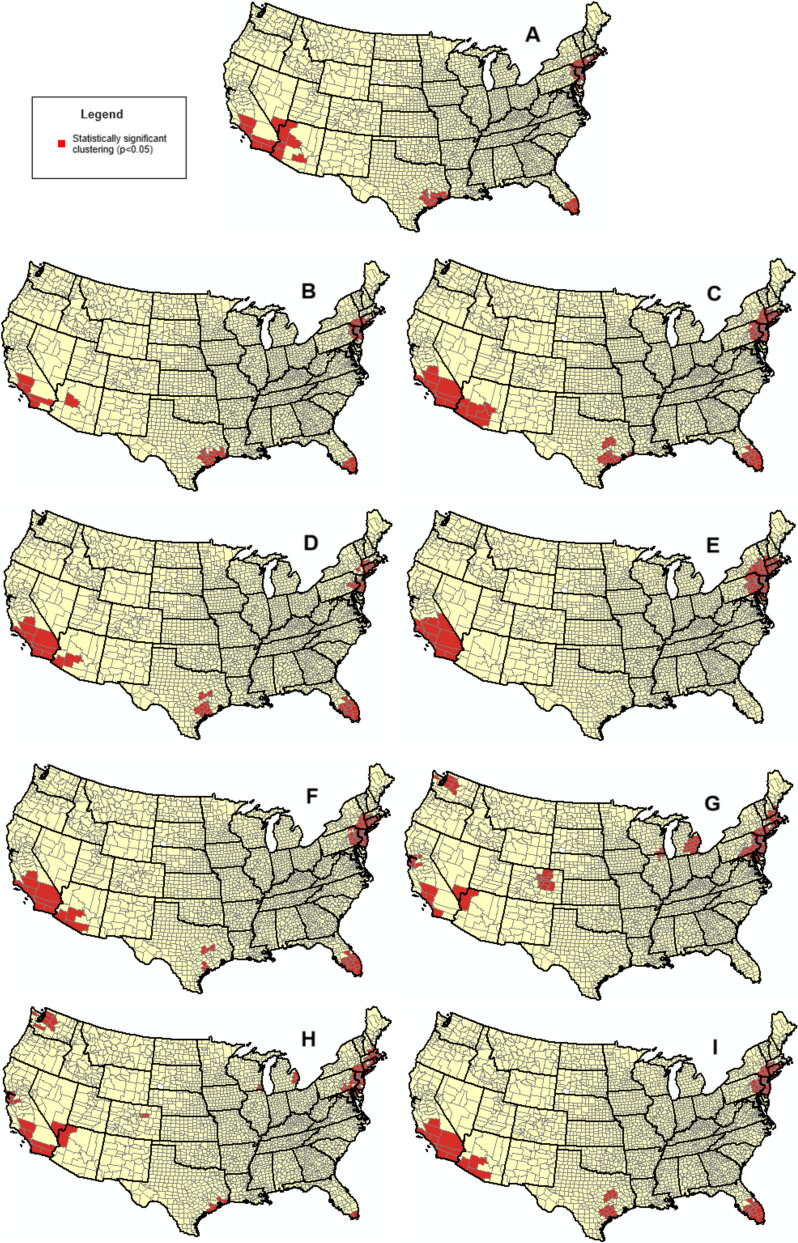
Clustering of COVID-19 study sites in the United States for (**A**) all studies, (**B**) Phase 1 trials, (**C**) Phase 2 trials, (**D**) Phase 3 trials, (**E**) Phase 4 trials, (**F**) interventional studies, (**G**) observational studies, (**H**) government-sponsored studies, and (**I**) industry-sponsored studies. Red shading indicates statistically significant hot spots

There were a total of 37,043 distinct sites in the United States for COVID-19 studies registered on CT.gov with trials taking place in all 50 states and the District of Columbia. Statistical analysis of spatial clustering revealed that all study types exhibited statistically significant hot spots centered around the Los Angeles Basin and New York City, though the expansiveness of the cluster differed across study types (see Appendix Fig. 3). More broadly, we observed local hot spot clusters of trials detected in Southern California expanding to Southern Arizona for Phase 2 trials, Phase 3 trials, interventional studies, and industry-sponsored studies; and a Northeastern cluster expanding as far north as the Boston Metropolitan Area for Phase 2 trials, Phase 4 trials, observational studies, and government-sponsored studies. Statistically significant hot spots were also evident in the Texas Triangle for all types of studies except Phase 4 trials and observational trials, with the same pattern observed for South Florida. Furthermore, significant hot spots were observed for the Denver Metropolitan Area, the Seattle Metropolitan Area, and parts of Michigan for observational studies and government-sponsored trials. No statistically significant cold spots were observed nationally (see Fig. 1).

When assessing potential demographic associations with trial location clustering at the national level, statistically significant relationships with race and ethnicity, sex, and age category covariates were evident in multivariable linear modeling for all types of normalized study counts (see Table 2). For all study types, either male residents per capita exhibited a significantly negative relationship or female residents per capita exhibited a significantly positive relationship, revealing a consistent sex-based pattern associated with proximity to a trial site. Furthermore, the normalized number of residents aged 25–34 years was significantly positively associated to the normalized rate of COVID-19 studies, for all study types. Conversely, the normalized number of residents aged 35–44 years was negatively related to the normalized rate of Phase 1 trials and interventional studies, while not being significantly associated with other study types.

**Table 2:** Multivariate models of COVID-19 studies in the United States for all studies, Phase 1 trials, Phase 2 trials, Phase 3 trials, Phase 4 trials, interventional studies, observational studies, government-sponsored studies, and industry-sponsored studies

Covariate	All Studies		Enrollment		Phase 1		Phase 2		Phase 3		Phase 4		Interventional		Observational		Govt Sponsored		Industry Sponsored	
	**β**	***p***	**β**	***p***	**β**	***p***	**β**	***p***	**β**	***p***	**β**	***p***	**β**	***p***	**β**	***p***	**β**	***p***	**β**	***p***
(Constant)	177	*0.012*	−159496	0.965	5	0.490	88	*0.006*	123	*0.006*	3	0.417	175	*0.004*	12	0.577	42	0.174	143	*0.004*
Black or African American	−11	0.593	−2011697	0.062	−2	0.463	20	*0.035*	4	0.777	−1	0.359	13	0.473	−25	< *0.001*	−27	*0.003*	17	0.241
American Indian/Alaska Native	180	< *0.001*	7655854	0.001	8	0.081	62	*0.002*	140	< *0.001*	3	0.142	195	< *0.001*	−1	0.960	28	0.152	149	< *0.001*
Asian	926	< *0.001*	37773918	< *0.001*	62	< *0.001*	293	< *0.001*	411	< *0.001*	25	< *0.001*	704	< *0.001*	189	< *0.001*	275	< *0.001*	501	< *0.001*
Native Hawaiian and Other Pacific Islander	−616	0.086	−21308532	0.253	−25	0.520	−200	0.221	−242	0.284	−18	0.311	−450	0.144	−174	0.112	−326	*0.038*	−258	0.313
Hispanic or Latino	103	*0.009*	2455695	0.232	7	0.107	59	*0.001*	80	*0.001*	0	0.828	111	*0.001*	−6	0.610	21	0.219	74	*0.009*
Other Race	−89	0.469	2801764	0.661	−4	0.746	−71	0.203	−63	0.419	2	0.808	−66	0.529	5	0.886	−25	0.646	−15	0.865
Multi-Racial	−749	*0.002*	−34051155	0.008	−56	*0.036*	−268	*0.017*	−438	*0.005*	−22	0.070	−685	*0.001*	−111	0.139	−198	0.067	−500	*0.004*
Male	−1268	< *0.001*	−11535128	0.502	−42	0.242	−564	< *0.001*	−628	*0.003*	−38	*0.021*	−1010	< *0.001*	−317	*0.002*	−555	< *0.001*	−748	*0.001*
Female	612	0.080	35979780	0.048	67	0.079	114	0.475	379	0.086	−2	0.911	575	0.056	47	0.659	48	0.755	448	0.072
Age 0–4 Years	−2001	*0.005*	−41789415	0.254	−264	*0.001*	−718	*0.025*	−971	*0.029*	−23	0.523	−1743	*0.004*	−273	0.203	−392	0.203	−1231	*0.014*
Age 5–9 Years	−1113	0.198	−127200877	0.005	28	0.767	−244	0.536	−986	0.070	−9	0.828	−1354	0.068	171	0.515	−111	0.769	−1115	0.070
Age 10–14 Years	1096	0.163	1584921	0.969	24	0.782	574	0.109	689	0.164	−4	0.913	1168	0.083	−172	0.472	417	0.225	594	0.288
Age 15–19 Years	−1181	*0.044*	18105192	0.552	−89	0.162	−367	0.170	−788	*0.033*	26	0.371	−1155	*0.022*	147	0.411	−375	0.143	−501	0.229
Age 20–24 Years	653	0.103	−51374090	0.014	−2	0.962	256	0.162	304	0.228	6	0.776	453	0.187	106	0.384	552	*0.002*	41	0.885
Age 25–34 Years	3365	< *0.001*	94000525	< *0.001*	210	< *0.001*	1310	< *0.001*	1542	< *0.001*	85	< *0.001*	2608	< *0.001*	739	< *0.001*	1109	< *0.001*	1960	< *0.001*
Age 35–44 Years	−972	*0.036*	−33958955	0.159	−104	*0.039*	−370	0.080	−505	0.084	5	0.839	−849	*0.033*	−61	0.665	−87	0.668	−586	0.076
Age 45–54 Years	356	0.320	−12107653	0.516	−21	0.590	283	0.084	96	0.671	16	0.374	222	0.471	200	0.067	318	*0.043*	127	0.618
Age 55–64 Years	250	0.637	−22617756	0.412	−12	0.830	172	0.477	82	0.807	24	0.367	103	0.822	171	0.290	316	0.173	26	0.946
Age 75–84 Years	−208	0.797	−4543545	0.914	−76	0.387	−12	0.974	−275	0.589	28	0.488	−265	0.702	105	0.669	357	0.312	−431	0.453
Age 85 + Years	640	0.306	−36063432	0.267	40	0.555	443	0.120	272	0.490	33	0.288	365	0.496	185	0.332	121	0.659	550	0.216

In regard to race and ethnicity, the number of Asian residents per capita was significantly positively related to proximity and enrollment of all types of trials. Both the number of American Indian or Alaska Native residents per capita and the number of Hispanic or Latino residents per capita were also positively associated with the number of industry-sponsored studies, Phase 2 trials, Phase 3 trials, and interventional studies per capita. Conversely, the number of Black or African American residents per capita was significantly negatively associated with the rate of government-sponsored studies and observational studies while being positively associated with the rate of Phase 2 trials. Finally, the number of Native Hawaiian or Other Pacific Islander residents per capita was significantly negatively associated with the number of government-sponsored studies per capita.

The distribution of the directionality of statistically significant associations nationally was similar for normalized study enrollment as for normalized study site number. In other words, demographic groups more likely to reside in counties with higher numbers of study sites tended to also reside in counties whose studies had higher enrollment. However, interestingly, despite the positive association of Hispanic or Latino residents with numerous study types, there was no significant relationship between overall county-level enrollment per capita and Hispanic or Latino residents per capita. Furthermore, though no significant relationships for the 20–24 years age group was observed with study site count, a statistically significant inverse association was observed with county-level enrollment.

### Localized hot spot analysis

**Fig. 2:**
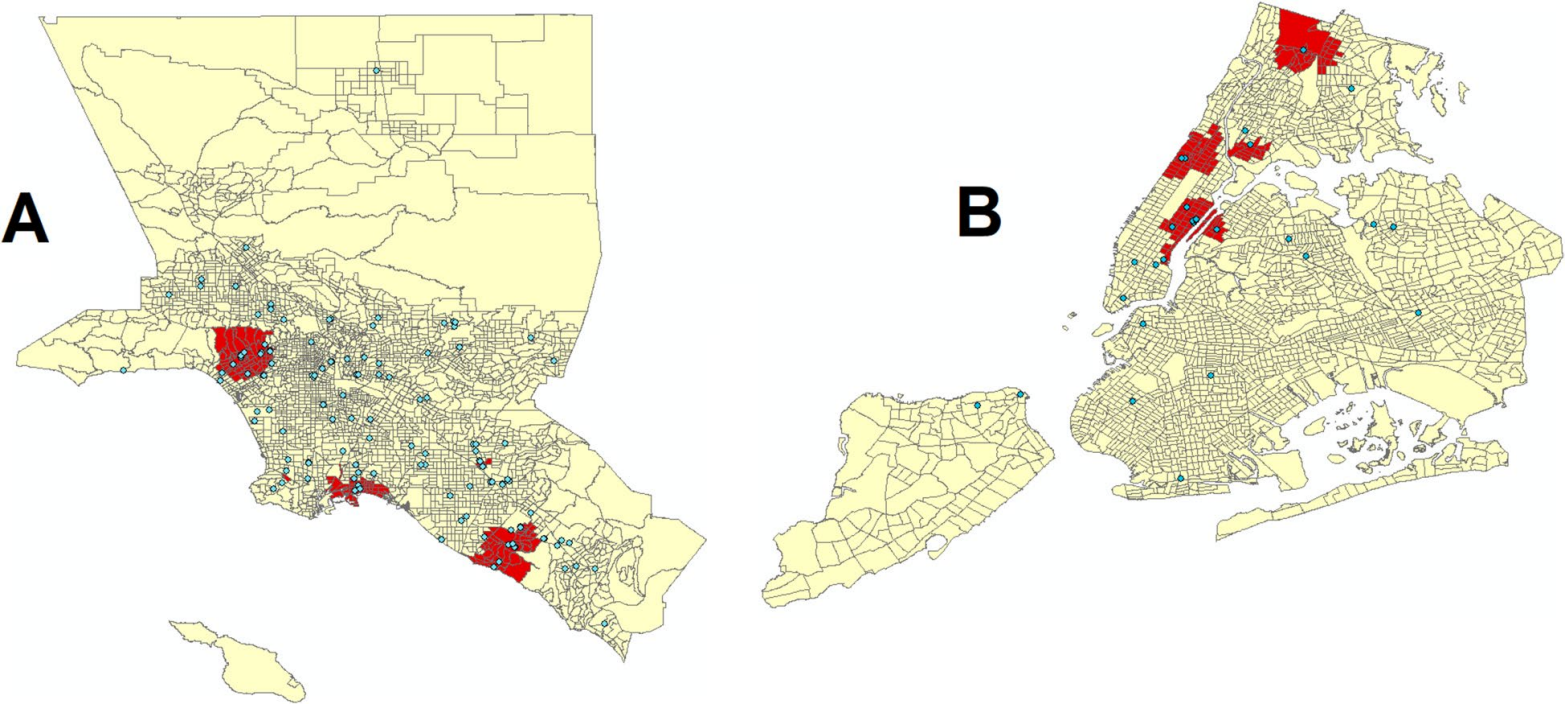
Clustering of COVID-19 study sites in (**A**) Los Angeles Basin and (**B**) New York City. Red shading indicates statistically significant hot spots and blue points represent location of individual trial sites

In the context of the two localized hot spots detected in our national geospatial analysis, 377 study sites (corresponding to 243 studies) were in the Los Angeles Basin (see Fig. 2A). Statistically significant hot spots in this area were observed in the Westwood neighborhood of Los Angeles, the coastal areas of Long Beach, and the city of Irvine. These local clusters appear to have been driven by, respectively, healthcare centers of the University of California, Los Angeles; Long Beach Medical Center and Long Beach Memorial; and healthcare centers of the University of California, Irvine. Pearson’s correlation coefficients computed between clustering *z*-scores and race and ethnic concentration at the census tract level revealed a significant positive association of clinical trial sites with density of non-Hispanic White residents per capita (*ρ* = 0.200, *p* < 0.001) and Asian residents per capita (*ρ* = 0.037, *p* = 0.037); no association with Native Hawaiian or Other Pacific Islander residents per capita; and significant negative associations with Black or African American residents per capita (*ρ* = −1.01, *p* < 0.001), American Indian or Alaska Native residents per capita (*ρ* = −0.168, *p* < 0.001), and Hispanic or Latino residents per capita (*ρ* = −0.267, *p* < 0.001; see Table 3A).

**Table 3:** Bivariate correlations between demographic covariates for (A) Los Angeles Basin (*n* = 2,958 census tracts) and (B) New York City (*n* = 2,170 census tracts). Emphasis added for statistically significant correlations

**Panel A**			**Panel B**		
**Covariate**	**Pearson's Rho**	***p***	**Covariate**	**Pearson's Rho**	***p***
White	0.2	< *0.001*	White	0.079	< *0.001*
Black or African American	−0.101	< *0.001*	Black or African American	−0.057	0.007
American Indian or Alaska Native	−0.168	< *0.001*	American Indian or Alaska Native	−0.013	0.536
Asian	0.037	*0.045*	Asian	−0.029	0.170
Native Hawaiian and Other Pacific Islander	0.033	*0.071*	Native Hawaiian and Other Pacific Islander	0.004	0.838
Hispanic or Latino	−0.267	< *0.001*	Hispanic or Latino	0.003	0.873
Other Race	−0.23	< *0.001*	Other Race	−0.008	0.704
Multi-Racial	0.057	*0.002*	Multi-Racial	0.003	0.875
Male	−0.051	*0.006*	Male	−0.047	0.030
Female	0.012	0.526	Female	0.057	0.008
Age 0–4 Years	−0.191	< *0.001*	Age 0–4 Years	−0.118	< *0.001*
Age 5–9 Years	−0.223	< *0.001*	Age 5–9 Years	−0.178	< *0.001*
Age 10–14 Years	−0.27	< *0.001*	Age 10–14 Years	−0.192	< *0.001*
Age 15–19 Years	−0.135	< *0.001*	Age 15–19 Years	−0.091	< *0.001*
Age 20–24 Years	0.06	*0.001*	Age 20–24 Years	0.097	< *0.001*
Age 25–34 Years	0.107	< *0.001*	Age 25–34 Years	0.164	< *0.001*
Age 35–44 Years	−0.018	0.330	Age 35–44 Years	0.029	0.173
Age 45–54 Years	−0.045	*0.014*	Age 45–54 Years	−0.073	0.001
Age 55–64 Years	0.065	< *0.001*	Age 55–64 Years	−0.015	0.488
Age 65–74 Years	0.119	< *0.001*	Age 65–74 Years	0.083	< *0.001*
Age 75–84 Years	0.123	< *0.001*	Age 75–84 Years	0.013	0.549
Age 85 + Years	0.16	< *0.001*	Age 85 + Years	0.022	0.295

There were 399 COVID-19 study sites in New York City belonging to 260 unique studies (see Fig. 2B). Cluster analysis revealed three statistically significant hot spots in the Upper East Side, the Upper West Side, and the Bronx. These appear to have been driven by, respectively, Weill Cornell Medical Center, Columbia University Irving Medical Center, and Montefiore Medical Center. As with the Los Angeles Basin, clustering of COVID-19 studies in New York City was significantly positively associated with per capita non-Hispanic White residents (*ρ* = 0.079, *p* < 0.001) and significantly negatively associated with per capita Black or African American residents (*ρ* = −0.057, *p* = 0.007; see Table 3B). However, these correlations were much weaker, and no significant associations were observed with normalized Asian nor Hispanic/Latino residents. Sex and age category correlations mirrored those for the Los Angeles Basin, with a significant inverse association with males per capita (*ρ* = −0.047, *p* = 0.030) and the two most strongly correlated age categories being the two youngest: age 0–4 years (*ρ* = −0.118, *p* < 0.001) and age 5–10 years (*ρ* = −0.178, *p* < 0.001). Multivariable models of clustering score with population-normalized demographic covariates also revealed discrepancies across race groups as well as significant negative associations with Black or African American race and Hispanic or Latino ethnicity in the Los Angeles area, though the group of age covariates and the group of sex covariates exhibited little variation in directionality (Appendix Table 1).

## Discussion

Results from this study indicate that there appears to exist a high degree of spatial variability in the geographic locations of COVID-19 clinical trial study sites registered in CT.gov, both nationally and when examining local communities identified through hot spot analysis, and that these discrepancies appear to be related to the demographic composition of certain areas of the country. This study also sought to determine whether demographic associations vary by type of study, but this variation remains unclear.

Descriptive analyses suggest that COVID-19-related clinical trials are mostly comprised of interventional trials rather than observational studies. Furthermore, among interventional trials, the intervention most commonly studied was a drug rather than a biologic or other means of addressing COVID-19 (e.g., behavior change, surgical technique, medical device, etc.). The relatively high proportion of industry-sponsored studies in this sample suggests high activity of industry actors in pursuing and investing in R&D for COVID-19 MCMs and an emphasis on dealing with public health emergencies.

Spatial clustering of COVID-19 studies also appears to be partly driven by total population, as New York City and Los Angeles are the two most populous cities in the United States. That being said, stratification of studies by type revealed inconsistencies in spatial clustering, especially as it relates to the Texas Triangle and Southern Florida, which did not exhibit statistically significant clustering for Phase 4 and observational studies. A discrepancy in the spatial clustering based on study design type may reflect differences in where investments are being made for generating real-world evidence on COVID-19, including those related to post-market surveillance, though more analysis is needed. We also observed that the influence of government funding appears to be mixed, as a more distributed set of overall study clustering was observed when compared to industry-sponsored studies. Government-funded studies exhibited positive associations with normalized Asian population, like industry sponsored studies. Industry-sponsored studies also exhibited positively associations with normalized White or American Indian or Alaska Native residents.

Analysis of the two largest national clusters revealed that local clustering may have been driven by the activities of large medical centers, especially those affiliated with urban academic health systems. In both areas, COVID-19 studies were located in areas with higher proportions of non-Hispanic White residents, and conversely located farther away from areas that were predominantly comprised of Black or African American communities. Though the influence of spatial distribution on actual participant recruitment practices is outside of the scope of this study, findings suggest that special attention should be given to recruit Black or African Americans into clinical trials, given the general extra distance of study sites from areas with higher proportions of Black or African American residents as observed in some of the U.S. areas reviewed in this study [17]. The creation of satellite study sites in these areas may be particularly effective in remediating proximity-related access barriers. Additionally, negative geographical proximity associations were observed with the youngest age groups (as well communities with a high proportion of parents with younger children). It is unknown whether this is an artifact of the high cost of living near urban academic health systems or the focus of clinical studies primarily on adult study participants, and whether the overall frequency of COVID-19 studies on children may be low due to population access or other concerns (e.g., ethical issues, low rate of severe illness) [18, 19].

Importantly, inadequate representation and inclusivity in clinical studies represents a threat to the potential utility of clinical evidence generated and as needed to benefit underrepresented communities already disproportionately impacted by COVID-19. Prior studies analyzing non-COVID-19 participant-level trial data have also found variable representation of racial and ethnic minorities for different therapeutic areas at US sites [10, 20]. Findings from this study suggest geographic maldistribution exists with respect to proximity for different age, sex, and racial and ethnic minority populations in COVID-19 clinical studies. A high degree of local spatial variation appeared to be driven by the location of major academic hospitals. However, in national analysis, clustering of COVID-19 studies did not appear to occur in all areas with major academic hospitals. Participants in clinical trials should reflect the individuals who may one day need to rely on drugs, vaccines or devices to manage their illnesses. Geographic maldistribution and travel burden can be a notable barrier for a certain demographic’s ability to participate in clinical trials and should be considered in the design of future studies.

### Limitations

Results of this study reflect general trends in community-level areas, and therefore are subject to ecological fallacy, whereby results may not apply to specific trials in those areas. Moreover, many associations uncovered as part of this study may be moderated by unaccounted moderators, such as transportation or community perspectives on clinical science. Many covariates obtained from CT.gov, including location, are derived from user-generated inputs with limited standardization. Efforts were made to remove misspellings and vague attributions from local analyses, whose patterns were sensitive due to small sample sizes and the effect of city-level attribution rather than that for specific locations. Nevertheless, national analysis may be partly influenced by user-generated error. Furthermore, the outcome variables denoting trials in spatial areas may suffer from constraints on internal reliability due to time varying confounding, particularly as the primary areas of investigation may have shifted over the course of the COVID-19 pandemic or there may have been major changes in predominant modalities of data collection (e.g., remote trial monitoring). Funder data from CT.gov is also generated from user input but classified as “NIH,” “Industry,” or “Other,” without more detailed information provided for the “Other” category, thereby limiting the identification of site subset with disproportionate spatial unavailability to any given demographic. Enrollment was provided at the study level, rather than the site level, without additional information about the distribution of either anticipated or realized enrollments across sites, and so the full number of subjects was attributed to multiple locations for multi-site studies. Information about the reliability of projected enrollment values was not made available. In addition, demographic data for enrollees of studies included in this study were not available for subject-level analysis, nor were race/ethnicity-specific data for rates of dropout or loss to follow-up. This cross-sectional study sought to assess spatial patterns of COVID-19 studies in the United States from a sample derived from CT.gov, which may suffer threats to validity from self-selection bias, particularly as relates to observational, Phase 1, or behavioral studies, which are generally exempt from CT.gov registration requirements. Future studies assessing disparities in access to clinical trials should seek to focus on individual study design strata with longitudinal follow-up, ideally addressing mechanisms pertinent to each stratum that may remediate disparities in participation by mitigating corresponding challenges to study access.

## Figures and Tables

**Figure:**
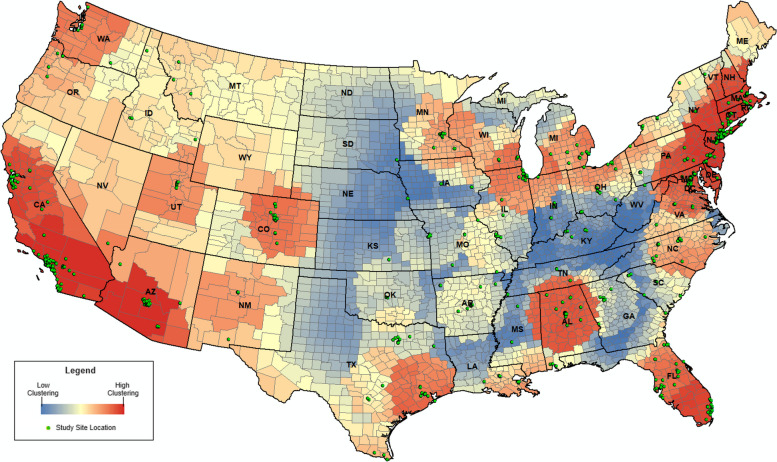
Locations of COVID-19 study sites (green points) overlaid above z-scores of the Getis Ord Gi* clustering statistic, with blue-yellow-red shading indicating low to high gradient of z-scores

## Data Availability

The datasets used and/or analyzed for the current study are available from the authors upon reasonable request.

## Appendix

**Table 4:** Results for a multivariate linear model between z-scores of clustering for all COVID-19 study sites with demographic covariates for (A) Los Angeles Basin (*n*=2,958 census tracts) and (B) New York City (*n*=2,170 census tracts). Emphasis added for statistically significant correlations

**Panel A**			** Panel B**		
**Covariate**	**β**	***p***	**Covariate**	**β**	***p***
Black or African American	-1.876	<0.001	Black or African American	-0.129	0.159
American Indian or Alaska Native	4.741	0.377	American Indian or Alaska Native	4.180	0.416
Asian	-0.969	<0.001	Asian	-0.329	0.040
Native Hawaiian and Other Pacific Islander	15.801	<0.001	Native Hawaiian and Other Pacific Islander	17.375	0.341
Hispanic or Latino	-3.606	<0.001	Hispanic or Latino	0.121	0.702
Other Race	3.968	<0.001	Other Race	0.307	0.630
Multi-Racial	0.801	0.657	Multi-Racial	-0.756	0.526
Male	3.603	0.014	Male	5.473	<0.001
Female	4.437	0.002	Female	7.864	<0.001
Age 0-4 Years	-8.270	0.002	Age 0-4 Years	-5.691	0.004
Age 5-9 Years	7.422	0.036	Age 5-9 Years	-7.725	0.001
Age 10-14 Years	-6.454	0.015	Age 10-14 Years	-13.973	<0.001
Age 15-19 Years	-4.321	0.002	Age 15-19 Years	-6.995	<0.001
Age 20-24 Years	-0.752	0.589	Age 20-24 Years	-3.763	0.008
Age 25-34 Years	0.602	0.670	Age 25-34 Years	-5.021	<0.001
Age 35-44 Years	-4.754	0.006	Age 35-44 Years	-6.919	<0.001
Age 45-54 Years	-8.951	<0.001	Age 45-54 Years	-7.319	<0.001
Age 55-64 Years	-2.658	0.195	Age 55-64 Years	-7.499	<0.001
Age 75-84 Years	-4.067	0.032	Age 75-84 Years	-6.773	<0.001
Age 85+ Years	0.673	0.761	Age 85+ Years	-9.169	<0.001
